# Potential Health Benefits of Yeast-Leavened Bread Containing LAB *Pediococcus pentosaceus* Fermented Pitaya (*Hylocereus undatus*): Both In Vitro and In Vivo Aspects

**DOI:** 10.3390/foods11213416

**Published:** 2022-10-28

**Authors:** Jacob Ojobi Omedi, Ning Li, Cheng Chen, Xin Cheng, Jing Huang, Binle Zhang, Tiecheng Gao, Li Liang, Zhongkai Zhou, Weining Huang

**Affiliations:** 1 State Key Laboratory of Food Science and Technology, Laboratory of Baking and Fermentation Science, Cereals/Sourdough and Nutritional Functionality Research, School of Food Science and Technology, Jiangnan University, Wuxi 214122, China; 2 Guangzhou Puratos Food Co. Ltd., Guangzhou 511400, China; 3 College of Food Science and Engineering, Tianjin University of Science and Technology, Tianjin 300457, China

**Keywords:** bread, pitaya, LAB fermentation, soluble dietary fiber, gut microbiota

## Abstract

This study aimed to investigate the effect of the incorporation of 0–25% pitaya (*Hylocereus undatus*) fermented by *Pediococcus pentosaceus* on physicochemical and bioactive properties of yeast-leavened wheat-mung bean bread. The results revealed that β-glucosidase activity increased during dough proofing, which may contribute to changes in dietary fiber. Compared to wheat bread, experimental bread had an increased content of soluble dietary fiber (SDF), total phenolic, total flavonoid, and slowly digestible starch, especially in wheat-mung bean bread prepared with 15% pitaya fermentates (WMB-15F). The effect of bread consumption on systemic inflammation, glucose tolerance, and blood lipid profiles was also evaluated via a mice model. The results indicated that levels of pro-inflammatory cytokines declined and glucose tolerance improved, while LDL and HDL were positively modified compared to control. Furthermore, an increased abundance of *Lactobacillus*, *Lachnospiraceae*, and *Bifidobacterium* spp. was observed in WMB-15F mice. Acetic acid was the dominant short-chain fatty acids (SCFAs) in feces and serum in all groups. Total SCFAs in circulation were highest in WMB-15F mice compared to other groups. In summary, an increased abundance of beneficial gut microbiota and promoted SCFA production might be highly associated with increased SDF and the release of key phenolic compounds during dough proofing, which exerts health benefits aroused from the consumption of yeast-leavened bread.

## 1. Introduction

Biotechnology has been increasingly applied in modern baking to the improve texture and nutritional aspects of bread [1,2]. Among them, sourdough fermented by defined lactic acid bacteria (LAB) starter together with baker’s yeast has attracted more interest in recent years [3,4], in which the metabolites achieved using LAB and yeast fermentation might be the major contributors [2]. 

Fruit-based substrates contain diverse health promoting molecules [5]; pitaya, an important popular tropical fruit, is commonly consumed [6]. Recent interest in pitaya studies has been fueled by the health promoting effects associated with its consumption [7,8,9]. Furthermore, previous studies also indicated that the fermentation of red pitaya pulp with probiotic strains *Lactobacillus acidophilus* LA-05 or *Bifidobacterium animalis* ssp. lactis BB-12 increased total phenolic compound content and antioxidant activity [10,11]. 

Similarly, the bioactive phenolic acid content of *Hylocereus undatus* substrate increased after fermentation with the selected antifungal LAB strains *Lactobacillus plantarum*, *Lactobacillus pentosus*, and *Pediococcus pentosaceus* [12]. These changes were suggested to be associated with LAB strains and/or species differences in releasing hydrolytic enzymes such as β-glucosidase during fruit substrate fermentation [13], because β-glucosidase (EC 3.2.1.21) is one of the most important LAB enzymes involved in the degradation of the β-(1→4) glucosidic linkages for enhancing the release of soluble dietary fiber (SDF) from insoluble dietary fiber (IDF) [14,15]. However, its effect on fruit-based dough and bread has been far from studied. 

In addition, studies on the effect of the intake of fruit-based yeast-leavened bread on gut microbiota and metabolite composition are also very limited. Therefore, a high β-glucosidase (5.51 U/mL) producing LAB *Pediococcus pentosaceus* isolated from Qu starters was applied in this study and its effect on the changes in the sugar composition, total phenolic (TPC), flavonoid (TFC) content, and phenolic acid composition of pitaya sourdough was investigated. Changes in β-glucosidase and α-amylase during dough proofing were analyzed to correlate enzyme activities with dough and/or bread quality properties. More importantly, the difference in gut microbiota composition and the production of SCFAs between a traditional white wheat bread and fermented pitaya (*Hylocereus undatus*) supplemented yeast-leavened bread was also evaluated via an animal model. Considering mung bean (*Vigna radiata* L.) as one of the most popular ingredients in foods, a portion of mung bean was also applied in the pitaya-supplemented sourdough.

## 2. Materials and methods

### 2.1. Materials, Chemicals and Microorganisms

Fifteen white pitaya (*Hylocereus undatus*) fruits were purchased at commercial maturity (~15 °Brix) from a local supermarket (Wuxi, China). Phenolic acid standards (caffeic, protocatechuic, ferulic, gallic, syringic, chlorogenic and p-hydroxybenzoic acids) and SCFAs (Acetic acid, propionic acid, butyric acid, iso-butyric acid, valeric acid, isovaleric acid) were purchased from Sinopharm Chemical Reagent Co., Ltd. (Beijing, China). Other chemicals used were of analytical grade. LAB strain *Pediococcus pentosaceus* (MW602529) was obtained from the Laboratory of Baking and Fermentation Science, Cereals/Sourdough and Ingredient Functionality Research, Jiangnan University.

### 2.2. Pitaya Fermentation and Bread Preparation

Pulp from selected pitaya was homogenized into a puree using a food blender. Puree (100 g) was weighed into a 250 mL Erlenmeyer flask, sealed and sterilized at 121 °C for 15 min, followed by cooling for 60 min. For use as inoculum, LAB *P. pentosaceus* was cultured in MRS broth at 30 °C for 24 h, followed by re-culturing to its late exponential phase (6 h) (cell concentration: 10^7^ CFU/mL). The cells were harvested by centrifugation at 8000× *g* for 15 min, then inoculated into the puree as starters. The pitaya puree was fermented at 30 °C for 24 h and used as sourdough in bread preparation.

Five types of bread, including wheat bread (Control), wheat-mung bean bread (WMB), wheat-mung bean bread incorporated with 5% (WMB-5F), 15% (WMB-15F) and 25% (WMB-25F) fermented pitaya, were applied. The recipe of the different breads is presented in Table S1. The bread was prepared following the procedure described by Omedi et al. [16]. Briefly, all weighed ingredients, except butter, were mixed in a spiral mixer (Sinmag, Wuxi, China) at slow speed (3 min), and fast speed (1 min). Butter was then added and mixed at slow speed (3 min) and fast speed (1 min). After mixing, dough was covered with polyethylene film and rested (5 min) at room temperature. Dough was then divided (90 g piece), rounded, and rested (5 min), followed by shaping and transfer into baking pans, then proofed (Sinmag, Wuxi, China) (90 min, 38 °C, 85% RH). This was followed by baking in a preheated oven (Sinmag, Wuxi, China) (top: 190 °C and bottom: 220 °C) for 21 min. After baking, the individual bread was cooled for 2 h at room temperature, used for further analysis and to prepare the feed formula for animal trials. 

#### 2.2.1. pH and Total Titratable Acidity (TTA) of Fermented Pitaya

The pH and TTA values of sourdough were determined in 10 g of sample homogenized with 90 mL of distilled water and measured using a pH meter (FE-20, Mettler Toledo, Shanghai, China). The TTA was expressed as mL of 0.1 N NaOH needed to achieve a pH of 8.5.

#### 2.2.2. Sugar Composition of Fermented Pitaya 

The sugar composition of samples was determined according to method described previously by Hernandez et al. [17]. Briefly, the sample (5 g) was mixed with 10 mL distilled water, then homogenized (200 rpm) in a constant temperature oscillation incubator, followed by centrifugation (10,000× *g*, 10 min, 4 °C). The supernatants (2 mL) were heated in a water bath (100 °C, 5 min), then cooled to room temperature, followed by centrifugation at 16,000× *g*, 30 min, 4 °C. The supernatant was then filtered (0.22 µm) for HPLC analysis using a XBridge BEH Amide column for separation. 

#### 2.2.3. Determination of Total Phenolic Content (TPC), Total Flavonoid Content (TFC), and Phenolic Acids of Fermented Pitaya 

Sample preparation: One gram of sample was extracted with 5 mL of methanol with constant shaking (200 rpm) for 24 h at 25 °C. The prepared extract was filtered and residue macerated with the same volume of fresh solvent, stirred and filtered. This was repeated three times. Finally, the extracts were filtered (0.22 μm filter), made up to 10 mL using the same solvent, and then stored for further analysis. 

The TPC in extracts was measured using the Folin–Ciocalteu method as described by Guergoletto et al. [18]. Absorbance was measured at 760 nm against distilled water as the blank prepared in the same way. Results were expressed as µg gallic acid equivalents/mL of sample (µg GAE/mL). The TFC in extracts was determined using a method described by a previous report [19], and the results were reported as mg rutin equivalent (RE) per g of the bread. 

Phenolic acids: A reverse phase HPLC method was used to identify and quantify phenolic acids in methanolic extracts of pitaya substrates before and after LAB fermentation as previously described Omedi et al. [12]. Analysis was carried out using the Waters HPLC system consisting of a Waters 1525 Binary HPLC pump, Waters 2707 Autosampler, and Waters 2489 UV–vis detector. Chromatographic separation was performed on a SYMMETRY C18 (5 μm, 4.6 × 250 mm) column. Elution was carried out with mobile phase (A): 1% aqueous acetic acid solution, mobile phase (B): acetonitrile. Flow rate: 0.7 mL/min, UV/Vis detector used 310 nm wavelength, and 35 °C column temperature. Elution gradient: 90% A (0–27 min), 10–40% B (28 min), 40–60% B (28–39 min), 60–90% B (39–50 min), then back to initial condition 90% A in 55 min. This was allowed to run for another 5 min, before the injection of another sample. Total analysis time per sample was 60 min. Methanol extracts were filtered (0.22 μm filter) prior to HPLC injection and 20 μL of sample was injected. Data acquisition and integration was performed using the Empower software package. The identification of phenolic acids in extracts was performed using external standards analyzed under similar conditions. 

#### 2.2.4. Determination of α-Amylase Activity and β-Glucosidase Activity in Dough

For the determination of enzyme activities, 1 g of dough samples were obtained before (0 min) and after (90 min) proofing was thoroughly mixed with deionized water (20 mL) and centrifuged (10,000× *g*, 15 min at 4 °C) to obtain supernatants that were used as substrates for the analysis. 

The β-glucosidase activity in dough was assayed by measuring the amount of p-nitrophenol (p-NP) liberated from 4-nitrophenyl β-D-glucopyranoside (p-NPG) as a substrate following a method previously described by Liang et al. [20] with some modifications. Briefly, to 100 μL of enzyme solution, 1.8 mL of 0.05 M sodium acetate (pH 5) buffer was added and incubated at 37 °C for 5 min, followed by adding 100 μL of substrate p-NPG or test substrate (supernatant) and incubation at 37 °C for 10 min. The reaction was stopped by adding 1 mL 1 M Na_2_CO_3_ and the absorbance of the yellow-colored p-nitrophenol was measured at optical density (OD) 400 nm. A standard curve using β-glucosidase enzyme (Shanghai Yuanye Biological Technology Co., Ltd., Shanghai, China) was used to determined enzyme activity. Each determination was performed in triplicates. Enzyme unit (U) was defined as the amount of enzyme required to catalyze the production of 1 μmol of p-NP per minute under the above conditions. 

The α-amylase activity was determined in dough using the DNS method described by Wang et al. [21] with some modifications. Briefly, supernatant (0.1 mL) from dough samples was added to soluble starch (0.9 mL) in the phosphate buffer and the mixture was incubated at 75 °C for 10 min. To the mixture, DNS solution (1%, *w*/*v*) was added followed by boiling for 10 min. The OD_540_ was measured. One unit of α-amylase activity was defined as the amount of enzyme required to release 1 mg of reducing sugar per minute. 

#### 2.2.5. Changes in the Dietary Fiber Content following the Dough Proofing

Dietary fiber composition, total dietary fiber (TDF), SDF (soluble dietary fiber), and IDF (insoluble dietary fiber) was determined using AACC method 32-07 [22]. 

#### 2.2.6. In Vitro Starch Digestibility of the Bread

After baking, the sample was sliced, the crust removed, and the crumb dried (45 °C for 5 h). After being homogenized and screened through a 0.5 mm sieve for obtaining a bread powder, its digestion property was determined as described by Rathod and Annapure [23] with some modifications. Briefly, 50 mg of bread in 0.2 M phosphate buffer, pH 6.9 was incubated in 0.5 mL of pancreatic amylase suspension (0.4 mg/mL of 0.2 M phosphate buffer, pH 6.9) at 20 °C for 2 h. An aliquot (0.1 mL) of sample was taken at 0, 20, and 120 min of incubation, and then 2 mL of 3,5-dinitrosalicylic acid reagent was added and the mixture was boiled for 5 min. The contents of the total starch (TS), rapidly digestible starch (RDS), and slowly digestible starch (SDS) in the bread were quantified using glucose content analyzed at 0 (FSG), 20 min (G20), and 120 min (G120) measured using the DNS method [23]. 

### 2.3. Customized Bread Diets, Animal and Study Design 

For use as diets in the animal study, specific volume (Control: 5.50 cc/g, WMB: 5.84 cc/g, WMB-5F: 5.51 cc/g, WMB-15F: 6.70 cc/g, WMB-25F: 5.45 cc/g) determined using the rapeseed displacement method was used as the bread quality selection criterion. Therefore, in this study, four types of breads including control, WMB, WMB-5F, and WMB-15F were selected for preparing the diets for the animal study. 

#### 2.3.1. Preparation of Customized Bread Diet

Customized bread diets were prepared by mixing the respective pulverized bread powders with the standard chow diet (AIN-93G) in a 1:1 ratio. To meet the Specific Pathogen Free (SPF) standards, the mixtures were processed into pellets, purified (irradiated), and vacuum packed by Jiangsu synergy pharmaceutical Bioengineering Co., Ltd. (Nanjing, China) (order number: xd202112001640) (Nutrition of diets presented in Table S2).

#### 2.3.2. Animal Study Design 

A total of 20 female 8-week-old C57BL/6J strain mice purchased from Vital River Laboratories (Beijing, China), were kept under a controlled temperature (22 ± 2 °C) on a 12 h light/12 h dark cycle in an SPF animal room. In this environment, the mice were acclimatized for 7 days during which they were fed on the standard chow diet (AIN-93G) and water ad libitum for 6 days followed by a 24 h fasting period on the seventh day with ad libitum water access. An initial fresh basal fecal sample was collected prior to the 24 h fast and stored at −80 °C for further analysis. After acclimatization, the mice were randomly allocated into four groups (*n* = 5) in separate cages, and each group allocated to a different diet. The mice in separate cages were then fed on their respective diet ad libitum for 21 days with ad libitum water access. On day 21, blood samples were collected from the orbital venous plexus of the eyes of each mouse after an overnight fasting, and then the mice were killed by decapitation. The animals were maintained and handled in accordance with the guidelines of the Ethics committee involving use of animal in Jiangnan University which approved this study (Reference number: JN. No202115c0450124[523]).

### 2.4. Determination of Changes in Gut Microbiota Composition

Fecal samples collected at day 0 (basal) and the 21 days of the experiment were used to analyze changes in gut microbiota composition. Microbial genomic DNA was extracted from frozen feces using TransGen AP221-02: TransStart FastPfu DNA polymerase Kit (TransGen Biotech, Beijing, China). The V3 + V4 region of the 16S rRNA was amplified by PCR and sequenced using an Illumina Hiseq2500 PE300 platform. The 16S rRNA gene sequencing was completed using Majorbio Bio-pharm Technology Co., Ltd. (Shanghai, China). 

### 2.5. Analysis of the Short-Chain Fatty Acids (SCFAs) Content in Feces and Serum

The content of SCFAs was determined in the feces and serum collected from mice groups as described previously [24,25] with some modification as follows: 

SCFAs in feces: briefly, 50 mg of the fecal sample was homogenized in Milli-Q water (1 mL) for 3 min, then kept at room temperature for 10 min, followed by centrifugation at 21,475× *g* for 10 min at 4 °C. The supernatants were then filtered through the 0.45 µm cellulose acetate filters and 400 µL filtrates were mixed with 100 µL of 50 µmol/mL internal standard (2-Ethylbutyric acid) solution, 10 µL of formic acid, and 490 µL of Milli-Q water in polypropylene vials. This was followed by centrifugation at 12,000× *g* for 15 min at 4 °C. From this, 700 µL aliquots of the supernatant were collected for SCFA analysis. 

SCFAs in serum: 200 μL of thawed serum (200 μL) was gently vortexed for 5 s, followed by the addition of 10 μL of formic acid, and 490 µL of Milli-Q water in polypropylene vials. This was followed by centrifugation at 12,000× *g* for 15 min at 4 °C, and then 700 µL aliquots of the supernatant (organic phase) were collected for SCFA analysis. A GC (Clarus 680 Gas Chromatography, PerkinElmer, Inc. Santa Clara, CA, USA) equipped with a HP-INNOWAX column (30 m × 0.250 mm × 0.25 µm, Agilent Technologies Inc., Santa Clara, CA, USA) was applied for the analysis, and helium was used at a flow rate of 1 mL/min. 

### 2.6. Analysis of the Bio-Parameters in the Serum Samples

The bio-parameters in the serum sample in terms of triglyceride (TG), total cholesterol (CHO), high-density lipoprotein (HDL) cholesterol, low-density lipoprotein (LDL) cholesterol, tumor necrosis factor-α (TNF-α), interleukin-1β (IL-1β), and interleukin-6 (IL-6) were determined, respectively, using Sbjbio mouse ELISA kits (SenBeiJia Biological Technology Co., Ltd., Nanjing, China). 

### 2.7. Statistical Analysis

Data were presented as the mean ± SEM, unless otherwise indicated. A Pearson correlation test was performed to explore associations between enzyme activities and dough and/or bread properties, and association between SCFAs and pro-inflammatory cytokines in serum. Statistical analysis was conducted using GraphPad Prism, version 9.2 (San Diego, CA, USA). Data were compared by one way analysis of variance (ANOVA), and a comparison between groups was conducted via a Tukey test. Significant differences were considered when *p* < 0.05. 

## 3. Results and Discussion

### 3.1. Effect of the Fermentation on Sugar and Phytochemical Content of the Pitaya Substrate 

Results for pH, TTA, sugar composition, TPC, TFC, and phenolic acids of the fermented pitaya substrate are presented in Table 1, in which the pH (4.89) and TTA (6.5 mL) values of pitaya substrate significantly (*p* < 0.05) declined (3.74 mL) and increased (7.3 mL), respectively, after fermentation. The content of glucose and maltose increased (*p* < 0.05), while fructose decreased in the pitaya substrate after LAB fermentation. In the previous report, red pitaya pulp fermented by probiotic LAB strains (*L. acidophilus* LA-05, *B. lactis* BB-12) and the glucose content was reported to decline, while maltose increased after fermentation [10]. The changes in sugar might be attributed to enzyme activities such as β-glucosidases from LAB strains fermentation, which hydrolyzed the glycosidic bond releasing the sugar moiety (e.g., glucose and maltose) from their conjugated precursor molecules [26]. In this study, the decrease of fructose content may imply that the fructose was the preferred carbon source for LAB during pitaya substrate fermentation. 

However, a general decrease of sugar content has been reported in several LAB fermented fruit substrate systems, especially over longer fermentation durations [11]. In the study, the short fermentation time (24 h) and the β-glucosidase producing LAB strain starter used might have contributed to the sugar composition during pitaya substrate fermentation. 

Furthermore, LAB fermentation significantly promoted the bio-functional potential of pitaya substrate, in which the content of bioactive phytochemicals, represented by TPC and TFC, significantly increased (*p* < 0.05) in pitaya after fermentation. In addition, the content of ferulic acid and p-hydroxybenzoic acid declined after pitaya fermentation. This indicated that ferulic acid, an important phenolic acid may have been decarboxylated during LAB fermentation of pitaya into other intermediate volatile compounds such as 4-vinylguaiacol. However, gallic acid, protocatechuic acid, chlorogenic acid, caffeic acid, and syringic acid were only detected after pitaya fermentation (Table 1). These findings were ascribed to the effect of LAB fermentation on the release of bound or conjugated flavonoids (TFC) and/or phenolics (TPC) (Table 1). 

### 3.2. Bioactive Components of the Dough and Its Corresponding Bread Supplemented with Pitaya Fermentates 

#### 3.2.1. Beta-Glucosidase and α-Amylase Activity in Dough 

The difference in the β-glucosidase and α-amylase activity before and after proofing is presented in Table 2. Compared to control, the β-glucosidase activity was higher in WMB, WMB-15F, and WMB-25F before proofing. The β-glucosidase activity values significantly declined in WMB, WMB-15F (*p* > 0.05), and WMB-25F, and increased (*p* < 0.05) in WMB-5F after proofing. However, compared to control, the β-glucosidase activity significantly increased in WMB-15F and WMB-25F, but decreased in WMB and WMB-5F. Moreover, in contrast with a recent study where doughs were enriched with LAB fermented kiwifruit substrates [20], the β-glucosidase activity in our study was two-fold higher before and after proofing. On the other hand, compared to control, the α-amylase activity was significantly increased after proofing with an increased concentration of pitaya fermentates. Furthermore, the β-glucosidase and α-amylase activities were positively correlated to the SDF, SDS, and TFC content of dough after proofing (Figure S1). Therefore, the changes in enzyme activities during proofing indicated bio-transformations of dough components such as starch and dietary fibers resulting in the release of conjugated bioactive molecules. 

#### 3.2.2. Difference in the Dietary Fiber Content among the Dough Samples

The results of TDF, IDF, and SDF in dough are presented in Table 2. Compared to control, TDF was the highest in WMB-25F and WMB-15F, followed by WMB-5F and WMB. Except WMB-25F, the TDF content was reduced in all doughs after proofing. The highest (*p* < 0.05) reduction was observed in control (−68.25%) and WMB (−59.56%) than in WMB-5F (−29.59%) and WMB-15F (−21.45%). Interestingly, the content of SDF increased (*p* < 0.05) in WMB-25F (89.60%), WMB-15F (84.69%), and WMB-5F (61.35%) than in WMB (48.02%) and control (16.45%) after proofing. The changes in dietary fiber might be attributed to β-glucosidase enzyme activity during proofing, which degraded β-(1→4) glucosidic linkages of the IDF component of TDF into more water-soluble polysaccharides and SDF [15]. More importantly, this study found that the SDF content was in the order WMB-25F > WMB-15F > WMB-5F > WMB > control. 

#### 3.2.3. In Vitro Starch Digestibility of the Bread 

The current study indicated that the total starch (TS) content of bread ranged from 213.19 to 424.57 mg/100 g, with the highest and lowest content observed in control and WMB, respectively (Table 2). Rapidly digestible starch (RDS) content followed a similar trend as TS, but slowly digestible starch (SDS) content increased in breads with increased concentration of pitaya fermentates. Changes in starch digestibility were attributed to dietary fiber, phenolic compounds, protein content, starch type, and the extent of starch gelatinization in the different breads [27]. 

#### 3.2.4. Difference in the Bioactive Components among the Breads

Total sugar content: Compared to control (7.09 mg/g), total sugar content increased (15.18–29.79%) in all breads, with a higher content in WMB-15F than WMB-5F, WMB-25F, and WMB (Table 2). The LAB fermentation of pitaya substrate increased (*p* < 0.05) the total sugar, glucose, maltose, and sucrose content (Table 1). Hence, the addition of pitaya fermentates gradually increased the total sugar content of bread. 

Total phenolic content (TPC): No significant difference was observed in the TPC content of all breads (Table 2). However, relative to control and WMB, the TPC content showed a tendency to be higher and lower, respectively, in the presence of pitaya fermentates. 

Total flavonoid content (TFC): On the other hand, compared to control (2.27 mg RE/g), TFC generally increased in WMB (0.44%), WMB-5F (7.80%), WMB-15F (11.62%), and WMB-25F (10.25%) (Table 2), showing an enhanced health benefit [5]. 

Soluble dietary fiber content: The SDF content was highest in WMB-15F, followed by control and WMB, and least in WMB-25F and WMB (Table 2). Previous studies have suggested that increased SDF content was effective at binding and complexing with polyphenols that increased their bioaccessibility and bioactivity potential [28]. Changes in TPC and TFC in this study were attributed to a dose dependent increase in adsorption and binding of phenolics to SDF to form a fiber-phenolic complex during dough/bread preparation. As a result, the supplementation of pitaya fermentates, especially at a 15% rate (WMB-15F) provided an optimal SDF content that interacted and bonded with released polyphenols (TFC, TPC) such as gallic acid, caffeic acid, and p-hydroxybenzoic acid to form several fiber-polyphenol complexes in dough and bread. In line with other published reports [28], we proposed that the intake of the fiber-polyphenol complex in bread may affect gut microbiota composition and promote health properties based on single (fiber or polyphenol) or synergistic (fiber-polyphenol) prebiotic interactive effects in a mice animal study model.

### 3.3. Effect of Diet Based on Bread Supplemented with Fermented Pitaya on Gut Microbiota in Mice

The Nonmetric multidimensional scaling (NMDS) plots of changes in the community of gut microbiota at basal and after 21 days of feeding on bread diets was shown in Figure S2a. Significant differences in the community structure of gut microbiota were observed before and after feeding mice on bread diets (Figure S2a). Compared to the gut microbiota at basal, the intake of pitaya containing diets, especially WMB-15F followed by WMB-5F significantly increased the α-diversity, evenness, and richness indices in the community structures of gut microbiota (Figure S2c–e). Additionally, the β-diversity of gut microbiota showed distinct and separate clustering patterns in the gut microbiota structures of the different mice groups (Figure S2b).

**Figure 1:**
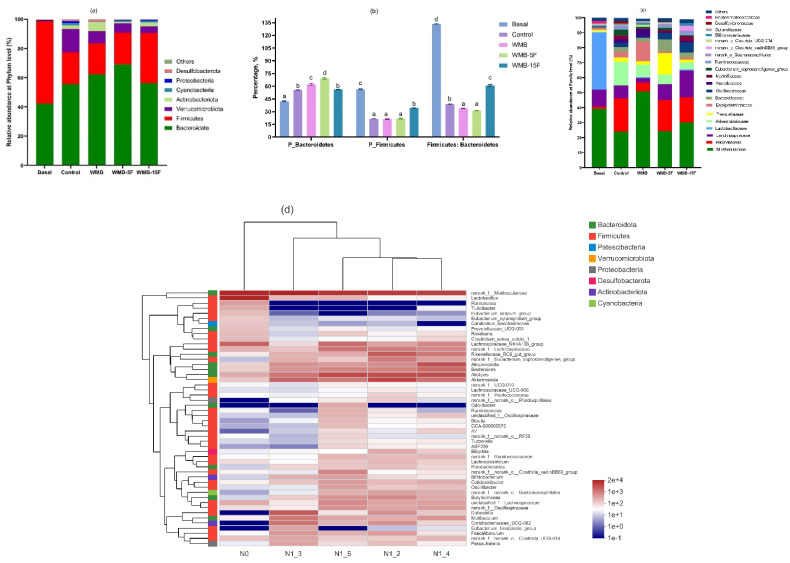
The relative abundance of gut microbiota at the phylum level (**a**) and family level (**b**) in mice groups at 0 (basal) and after 21 days fed on customized bread diets. The relative abundance of *Firmicutes*, *Bacteroidetes*, and *Firmicutes*: *Bacteroidetes* ratio (**c**). Heat map cluster analysis of the effect of customized bread diets on mice groups at 0 (basal, N0) and after 21 days fed on control (N1_2), WMB (N1_3), WMB-5F (N1_4), and WMB-15F (N1_5) (**d**). Different lower-case letters in the same column and upper-case letter in same row for specific treatment indicated significant difference (*p* < 0.05).

Furthermore, the results of the effect of a sourdough-containing bread diet intake on gut microbiota before (basal) and after 21 days of feeding are presented in Figure 1a–d. At phyla level, compared to the basal, a reduction of the relative abundance of the main phylum *Firmicutes* and *Bacteroidetes* was observed after the intake of bread, with a lower reduction observed in WMB-15F and WMB-5F fed mice compared to control and WMB (Figure 1a). Compared to control, the relative abundance of *Bacteroidetes* and *Firmicutes* increased and decreased, respectively, in mice fed on a fermented pitaya-containing bread diet. However, mice fed on WMB-15F had significantly increased *Firmicutes* and decreased *Bacteroidetes* proportions than the WMB-5F, WMB, and control mice. In line with that observations that a healthy gut is reported to consist of two main phyla of *Firmicutes* and *Bacteroidetes* that contribute to a 90% relative abundance [29], intake of the fermented pitaya-containing bread diets restored the relative abundance of the main phylum compared to control and WMB. 

At the family level, an increase of the relative abundance of *Muribaculaceae*, *Rikenellaceae*, *Lanchnospiraceae*, *Akkermansiaceae*, *Prevotellaceae*, and *Erysipelotrichaceae* was observed in all mice groups fed on the different bread diets (Figure 1c). However, the relative abundance of *Lanchnospiraceae*, *Lactobacillaceae*, *Ruminococcaceae*, and *Bifidobacteriaceae* was significantly higher in WMB-15F fed mice groups (Figure 1c). *Muribaculaceae* and *Lanchnospiraceae* belonging to phyla *Bacteroides* and *Firmicutes*, respectively, were prevalent in the human gut and known to be specialized in the utilization of complex carbohydrates resulting in the production of SCFAs [30,31]. In addition, an increased relative abundance of *Lactobacillaceae*, *Ruminococcaceae*, and *Bifidobacteriaceae* was associated with higher prebiotic containing diets that promoted beneficial and healthy gut microbiota composition [29]. 

At genus level, compared to basal, the relative abundance of *Ruminococcus* and *Bifidobacterium* were only elevated (*p* ≤ 0.001) in WMB-15F (Figure 1d). Compared to control, levels of norank_f_*Muribaculaceae, Muribaculum, Dubosiella, Coriobacteriaceae* UCG 002, *Faecalibaculum, Parasutterella*, and norank_f_norank_*Clostridia*_UCG-014 were elevated (*p* ≤ 0.001) in WMB, while *Alistipes*, *Lanchnospiraceae NK4A136* group, and unclassified_f_*Lanchnospiraceae* were elevated (*p* ≤ 0.001) in WMB-5F and WMB-15F. Furthermore, compared to WMB, levels of *Alistipes*, *Bacteriodes*, *Lanchnospiraceae NK4A136* group, norank_f_*Lanchnospiraceae* in WMB-5F and WMB-15F, *Alloprevotella, Rikenellaceae* RC9, *Eubacterium_coprostanoligenes* group and norank_f_*Oscillospiraceae* in WMB-5F, unclassified_f_*Lanchnospiraceae*, norank_f_norank_*Clostridia*_vadinBB60 group, *Lactobacillus*, *Roseburia*, *Blautia*, and *Bifidobacterium* in WMB-15F were elevated (*p* ≤ 0.001). 

Changes in gut microbiota composition might be explained by the prebiotic effect due to the increased content and release of SDF and polyphenol (TFC, TPC)—such as gallic acid, caffeic acid, and p-hydroxybenzoic acid—in fermented pitaya-containing bread diets attributed to specialized enzymes (e.g., lyases, glycoside hydrolases and esterases) from the dominant gut microbiota that might have hydrolyzed and released SDF and phenolics from the fiber-polyphenol complexes in bread diets. Our findings were in agreement with an earlier study that found that, in a dose-dependent manner, SDF modulated the growth of beneficial gut microbiota such as *Bifidobacterium* [32]. Similarly, the intake of polyphenols increased growth of *Lactobacillus* spp., *Bifidobacterium* spp., and *Ruminococcus* spp., in an in vivo human study [33]. It is proposed that the promoted bioactive components (e.g., SDF and polyphenols) in the fermented pitaya wheat-mung bean dough/bread matrix played a prebiotic effect on gut microbiota, and therefore, a higher concentration of pitaya fermentates enhanced prebiotic properties of the bread and contributed to a healthier gut microbiota. 

### 3.4. Effect of Diet Based on Bread Supplemented with Fermented Pitaya on SCFAs Production in Serum and Feces of the Mice

**Figure 2:**
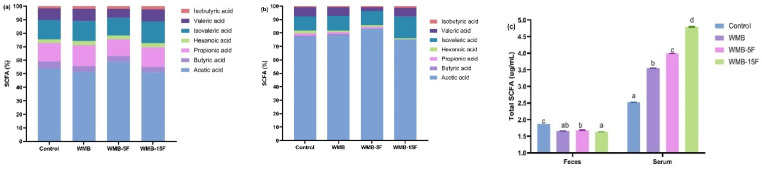
Relative content of short-chain fatty acids (SCFAs) in fecal (**a**) and serum (**b**) samples; total SCFA content (**c**) in mice fed with customized bread diets. Bars represented the mean ± SEM (*n* = 3). Different letters implied significant difference at *p* < 0.05.

The total content and composition of SCFAs in the serum and feces of mice are presented in Figure 2. Acetic acid was the dominant SCFA in feces (0.84–1.01 µg/mL) and serum (1.95–3.60 µg/mL) with relative abundances of 51.17–58.79% and 75.18–83.19%, respectively (Figure 2a,b), followed by propionic acid, isovaleric acid, valeric acid, butyric acid, hexanoic acid, and iso-butyric acid (Figure 2a), whereas in the serum they were isovaleric acid, valeric acid, butyric acid, and hexanoic acid (Figure 2b). Prebiotic enriched products such as those high in SDF and polyphenols provided adequate energy sources for obligate anaerobic bacteria whose fermentative activity resulted in the increased production of SCFAs [34]. The trend in total SCFAs in serum and feces was in agreement with the data from bioactive contents (soluble dietary fiber, phenolics) in bread (Table 2) for showing their prebiotic effect on gut microbiota composition (Figure 1). Therefore, it was hypothesized that the synergistic interaction between the increased SDF and phenolic contents in wheat-mung bean bread diets supplemented with pitaya fermentates could reduce the rate of starch digestibility that ensured the improvement of the distribution of carbon sources for gut microbiota fermentation along the entire length of colon. This resulted in increased SCFA production in the last part of the colon, followed by high absorption into systemic circulation where SCFAs such as acetic acid for providing health benefits in the order WMB-15F > WMB-5F > WMB > control.

### 3.5. Effect of Diet Based on Bread Supplemented with Fermented Pitaya on the Serum Levels of Pro-Inflammatory Cytokines 

**Figure 3:**
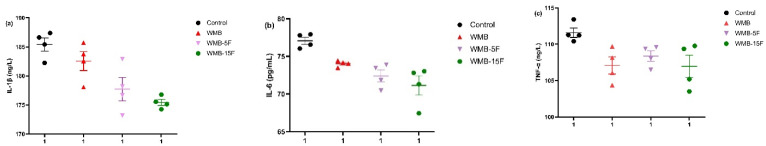
Effect of pitaya fermentate in bread diets on pro-inflammatory cytokines, including IL-1β (**a**), IL-6 (**b**), and TNF-α (**c**). Bars represented the mean ± SEM (*n* = 4).

The results of the effect of pitaya fermentates supplementation in yeast leavened wheat-mung bean bread diets on the serum content of the selected pro-inflammatory cytokines of IL-1β, IL-6, and TNF-α were presented in Figure 3. The wheat bread (control) group had the highest IL-1β (185.42 ng/L), IL-6 (77.08 pg/mL), and TNF-α (111.58 ng/L). Compared with control, the levels of IL-1β, IL-6, and TNF-α declined with the increased concentration of pitaya fermentates in wheat-mung bean bread (Figure 3a–c). The reduction of pro-inflammatory cytokines may be explained by the combined prebiotic effect of dietary fiber and phenolic compounds from pitaya fermentates.

Similar observations were reported in healthy mice after 21 days’ intake of a multi-cereal-sourdough bread relative to wheat bread [35]. In this study, the levels of pro-inflammatory cytokines were negatively correlated to total serum SCFAs and serum acetic acid content (r = −0.9, *p* = 0.05; r = −0.9, *p* = 0.05, respectively). Therefore, our findings indicated that the consumption of wheat-mung bean bread supplemented with pitaya fermentates promoted anti-inflammatory properties in mice. 

### 3.6. Effect of Diet Based on Bread Supplemented with Fermented Pitaya on Glucose Tolerance and Serum Lipid Profile

**Figure 4:**
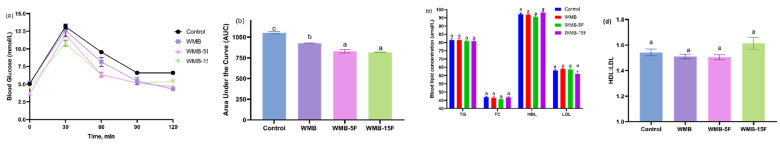
Effect of pitaya fermentate supplementation in bread diets on: (**a**) Glucose tolerance test of mice after 12 h fasting. (**b**) Average area under the curve (AUC) of glucose tolerance test of mice after 12 h fasting. (**c**) Lipid profile concentration, including TG, TC, HDL, LDL. (**d**) The HDL: LDL ratio in different mice. Bars represented the mean ± SEM (*n* = 5). Different letters implied significant difference at *p* < 0.05. TG: Triglyceride; TC: Total cholesterol; HDL: High-density lipoprotein; LDL: Low-density lipoprotein.

The effect of fermented pitaya-containing bread diet on the changes in glucose tolerance and lipid profile (TG, TC, HDL, and LDL) is shown in Figure 4. Although no significant differences were observed among the mice groups at the end of the fasting period (12 h) (Figure 4a), glucose levels were slightly lower (*p* ≥ 0.05) in WMB-5F and WMB-15F compared to control and WMB at the baseline. More importantly, following the injection of glucose, blood glucose levels significantly increased (*p* ≤ 0.05) in the first 30 min in the order control > WMB > WMB-5F > WMB-15F, followed by a rapid decline at 60 min and a slight levelling off at 90 and 120 min. Moreover, blood glucose levels in WMB-5F and WMB-15F decreased more rapidly compared to control and WMB at 60 min. The area under curve (AUC) was lowest in WMB-15F and WMB-5F followed by WMB and control (Figure 4b). This implied that a higher proportion of fermented pitaya-containing bread in the diet significantly improved glucose intolerance in mice. The mechanisms involved in the improvement might be attributed to a multifunction achieved by an enriched dietary fiber and phenolic content and even the gut microbiota metabolites. 

The supplementation of fermented pitaya in bread, especially at 15% (WMB-15F) had a tendency to lower LDL and increase HDL contents (*p* ≥ 0.05) in comparison to control and WMB, although the difference was not significantly distinct (Figure 4c). This may further suggest that a longer supplementation was required. Nevertheless, this resulted in a tendency of the HDL:LDL ratio to increase in WMB-15F fed mice (Figure 4d). 

## 4. Conclusions

Supplementation of pitaya substrate fermented by LAB increased the content of soluble dietary fiber and some key active components during dough proofing. Furthermore, dough/bread supplemented with pitaya substrate fermentate was also characterized and promoted the level of slowly digestible starch. In the in vivo study, the consumption of the bread, especially at 15% pitaya fermentate supplementation, altered the gut microbiota composition. In particular, a shift towards the increased abundance of beneficial health promoting bacteria such as *Lactobacillus*, *Lachnospiraceae*, and *Bifidobacterium* spp. resulted in the increased production of total SCFAs in mice. Moreover, the intake of sourdough-containing bread diets supplemented with 15% pitaya fermentates promoted anti-inflammatory properties, improved glucose intolerance, and increased the (*p* > 0.05) HDL: LDL profile in mice. These findings indicated the role played by β-glucosidases produced by carefully selected LAB strains from Qu starters on enriching pitaya substrates as novel ingredients in a fruit-based wheat-mung bean bread system. In line with consumer demand for health-promoting and clean-label products, this study contributed new insights on the LAB fermentation of fruit substrates for application in the baking food industry. 

## Figures and Tables

**Table 1:** Physicochemical properties, sugar composition and phenolic acid content in white pitaya substrate fermented by *P. pentosaceus*.

	Pitaya Substrate	
Characteristic	Before Fermentation	After Fermentation
pH	4.89 ± 0.06 b	3.74 ± 0.42 a
TTA (mL)	6.5 ± 0.07 a	7.3 ± 0.07 b
**Sugars (mg/mL)**		
Glucose	1.88 ± 0.01 a	2.26 ± 0.00 b
Fructose	5.95 ± 0.00 b	5.56 ± 0.00 a
Maltose	5.10 ± 0.02 a	6.40 ± 0.00 b
Total	12.93 ± 0.03 a	14.22 ± 0.00 b
**Phytochemical content**		
TPC (mg GAE/mL)	1.25 ± 0.20 a	1.51 ± 0.20 b
TFC (mg RE/g)	1.48 ± 0.04 a	1.66 ± 0.04 b
**Phenolic acids (100 µg/mL)**		
Gallic acid	-	2.37 ± 0.04
Protocatechuic acid	-	0.76 ± 0.03
Chlorogenic acid	-	0.52 ± 0.01
Caffeic acid	-	5 × 10^−4^ ± 2 × 10^−5^
Syringic acid	-	0.51 ± 0.01
p-hydroxybenzoic acid	3228.89 ± 0.15 b	2056.31 ± 0.30 a
Ferulic acid	6.19 ± 0.00 b	1.67 ± 0.00 a

Values were mean ± standard deviation (*n* = 3). Different letters in the same row indicated significant different (*p* < 0.05) according to Duncan’s test. Nd: not determined. (-): not detected. TTA: Total titratable acidity. TPC: Total phenolic compound. GAE: Gallic acid equivalent. TFC: Total flavonoid content. RE: Rutin equivalent.

**Table 2:** Effect of pitaya fermentate supplementation on enzyme activities, the dietary fiber composition of dough, bioactive components, and starch digestibility of bread.

	Dough Analysis										Bread Analysis						
	Enzyme Activity (U/g)				Dietary Fiber Composition (mg/g)						Bioactive Components				In Vitro Starch Digestibility (mg/100 g)		
	β-Glucosidase		α-Amylase		IDF		SDF		TDF		Total Sugars (mg/g Bread)	TPC (mg GAE/mL)	TFC (mg RE/g)	SDF (mg/g)	TS	RDS	SDS
	0 min	90 min	0 min	90 min	0 min	90 min	0 min	90 min	0 min	90 min							
Control	2.17 ± 0.03 aA	2.30 ± 0.01 bB	7.34 ± 0.73 aA	7.63 ± 0.14 aA	0.097 ± 0.001 a	0.027 ± 0.001 a	0.069 ± 0.000 b	0.025 ± 0.001 a	0.166 ± 0.001 b	0.053 ± 0.000 a	7.09 ± 0.08 a	1.65 ± 0.07	2.28 ± 0.01 a	0.043 ± 0.000 b	424.57 ± 11.78 d	411.54 ± 12.03 e	13.03 ± 0.25 a
WMB	2.47 ± 0.01 cB	2.20 ± 0.01 aA	8.42 ± 0.08 bA	8.57 ± 0.12 bA	0.105 ± 0.001 c	0.028 ± 0.001 a	0.073 ± 0.001 b	0.044 ± 0.005 b	0.178 ± 0.000 c	0.072 ± 0.005 b	8.16 ± 0.03 ab	1.83 ± 0.13	2.29 ± 0.04 a	0.043 ± 0.000 b	213.19 ± 24.09 a	197.84 ± 24.09 a	15.35 ± 0.00 a
WMB-5F	2.12 ± 0.01 aA	2.22 ± 0.01 aB	8.26 ± 0.19 bA	8.99 ± 0.01 cB	0.100 ± 0.000 b	0.051 ± 0.001 d	0.045 ± 0.002 a	0.051 ± 0.001 c	0.146 ± 0.002 a	0.103 ± 0.002 c	9.14 ± 0.54 b	1.74 ± 0.07	2.45 ± 0.01 b	0.045 ± 0.000 c	263.08 ± 2.12 b	247.37 ± 0.60 b	15.71 ± 1.51 a
WMB-15F	2.39 ± 0.03 bA	2.33 ± 0.02 cA	8.48 ± 0.07 bA	9.72 ± 0.11 dB	0.104 ± 0.001 c	0.037 ± 0.001 b	0.067 ± 0.001 b	0.097 ± 0.002 d	0.171 ± 0.001 c	0.134 ± 0.003 d	9.20 ± 0.91 b	1.71 ± 0.11	2.54 ± 0.03 b	0.046 ± 0.000 d	307.75 ± 17.01 c	285.61 ± 18.53 c	22.13 ± 1.51 b
WMB-25F	2.57 ± 0.05 dB	2.46 ± 0.00 dA	10.22 ± 0.06 cA	10.69 ± 0.20 eB	0.105 ± 0.000 c	0.042 ± 0.000 c	0.072 ± 0.005 b	0.142 ± 0.004 e	0.177 ± 0.004 c	0.184 ± 0.004 e	8.72 ± 0.16 b	1.79 ± 0.06	2.51 ± 0.11 b	0.042 ± 0.000 a	329.30 ± 0.78 c	294.67 ± 1.79 d	34.63 ± 1.01 c

Values were mean ± standard deviation (*n* = 3). Different lower-case letters in the same column and upper-case letter in same row for specific treatment indicated significant difference (*p* < 0.05). Where 0 min and 90 min represented start and end of dough proofing. WB: Wheat control dough/bread. WMB: Wheat-mung bean dough/bread. WMB-5F, WMB-15F, and WMB-25F: Wheat-mung bean dough/bread containing 5%, 15%, 25% pitaya substrate fermented by *P. pentosaceus*, respectively. IDF: Insoluble dietary fiber. SDF: Soluble dietary fiber. TDF: Total dietary fiber. TPC: Total phenolic compound. GAE: Gallic acid equivalent. TFC: Total flavonoid content. RE: Rutin equivalent. TS: Total starch. RDS: Rapidly digestible starch. SDS: Slowly digestible starch. N.B: The β-glucosidase enzyme activity of fermented pitaya was 3.68 U/mL.

## Data Availability

The data presented in this study are available on request from the corresponding author.

## Supplementary Materials

The following supporting information can be downloaded at: https://www.mdpi.com/article/10.3390/foods11213416/s1. Table S1. Formulation of bread for use in this study. Table S2. Formulation and nutrition of the different customized bread diets. Figure S1. Pearson correlation of enzyme activities and properties of dough and bread. Figure S2. The non-metric multi-dimensional scaling plot (NMDS) of the Bray–Curtis similarity coefficients calculated from the 16S rRNA (a). PcoA results on β-diversity of each group (b). Changes in α-diversity revealed by the Shannon index (c), evenness revealed by Shannoneven index (d), and richness revealed by sobs (e) of gut microbiota from different mice groups fed on customized bread diets.
