# Supplementary material for: Potential Health Benefits of Yeast-Leavened Bread Containing LAB Pediococcus pentosaceus Fermented Pitaya (Hylocereus undatus): Both In Vitro and In Vivo Aspects
Source: Foods. 2022 Oct 28;11(21):3416. doi: 10.3390/foods11213416 (PMC9653669; doi:10.3390/foods11213416)
Supplement: Supplementary file 1 [file foods-11-03416-s001.zip › foods-1929245-supplementary.pdf]

Supplementary material.

Table S1. Formulation of bread for use in this study.

	Sample ID				
**Ingredient (s)**	Control	WMB	WMB-5F	WMB-15F	WMB-25F
Wheat flour (g)	300	270	270	270	270
Mung bean flour (g)	-	30	30	30	30
Yeast (g)	6	6	6	6	6
Sugar (g)	30	30	30	30	30
Salt (g)	3	3	3	3	3
Margarine (g)	24	24	24	24	24
*Pitaya fermentate (g)	-	-	15	45	75
Water (mL)	180	186	159	136	113

*MC: 86%, Total dietary fiber: 0.2 mg/g (Insoluble dietary fiber: 0.14 mg/g, Soluble dietary fiber: 0.06 mg/g).

Table S2. Formulation and nutrition of the different customized bread diets.

	**Customized bread diets****			
**Ingredient (g)**	Control	WMB	WMB-5F	WMB-15F
Bread powder*	500	500	500	500
Casein, 30 Mesh	100	100	100	100
L-Cystine	1.5	1.5	1.5	1.5
Corn Starch	198.5	198.5	198.5	198.5
Maltodextrin	66	66	66	66
Sucrose	50	50	50	50
Cellulose	25	25	25	25
Soybean Oil	35	35	35	35
t-Butylhydroquinone	0.007	0.007	0.007	0.007
Mineral Mix S10022G	17.5	17.5	17.5	17.5
Vitamin Mix V10037	5	5	5	5
Choline Bitartrate	1.25	1.25	1.25	1.25
**Total**	1000	1000	1000	1000

*Bread (Table S1) dried (40 °C for 8 h) and pulverized into a powder. ** Processed into pellets, purified (irradiated) by Jiangsu synergy pharmaceutical Bioengineering Co., Ltd (Nanjing, China) (Order number: xd202112001640).

Figure S1. Pearson correlation of enzyme activities and properties of dough and bread.

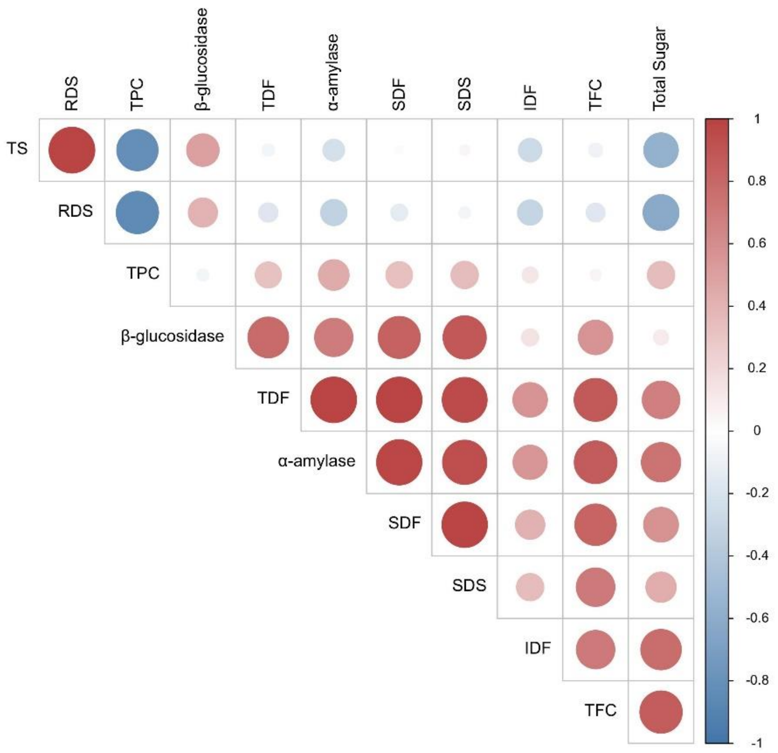

IDF: Insoluble dietary fiber. SDF: Soluble dietary fiber. TDF: Total dietary fiber. TPC: Total phenolic compound. TFC: Total flavonoid content. TS: Total starch. RDS: Rapidly digestible starch. SDS: Slowly digestible starch.

Figure S2. The non-metric multi-dimensional scaling plot (NMDS) of the Bray-Curtis similarity coefficients calculated from the 16S rRNA (a). PcoA results on β-diversity of each group (b). Changes in α-diversity revealed by the Shannon index (c), evenness revealed by Shannoneven index (d), and richness revealed by sobs (e) of gut microbiota from different mice groups fed on customized bread diets.

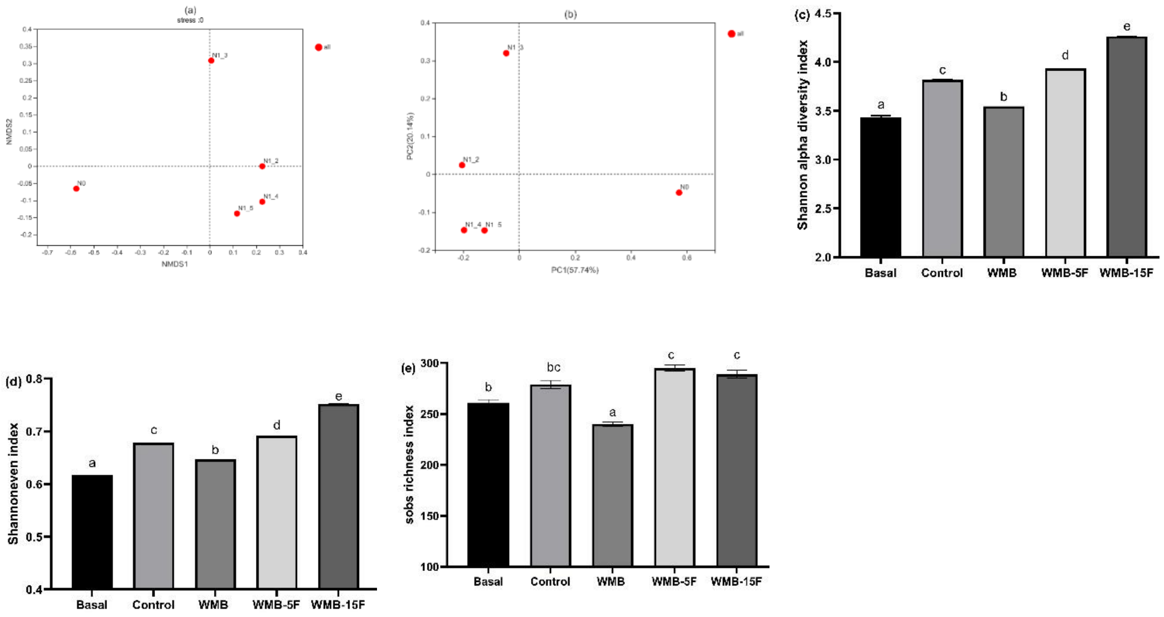

Mice groups at 0 (basal, N0) and after 21 days fed on control (N1_2), WMB (N1_3), WMB-5F (N1_4), and WMB-15F (N1_5).
